# Supplementary material for: A High Hepatic Uptake of Conjugated Bile Acids Promotes Colorectal Cancer—Associated Liver Metastasis
Source: Cells. 2022 Nov 28;11(23):3810. doi: 10.3390/cells11233810 (PMC9736302; doi:10.3390/cells11233810)
Supplement: Supplementary file 1 [file cells-11-03810-s001.zip › Supplementary materials_final.pdf]

FigureS1.

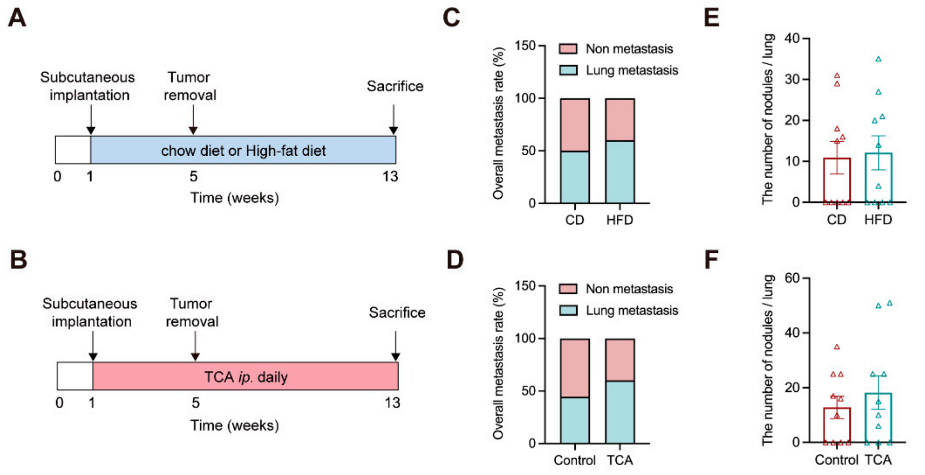

Figure S1. The effect of HFD or TCA on lung metastasis in a subcutaneous implantation mouse model. **(A, B)** The timeline of the animal experiments (n=10/groups); (C, D) The rate of lung metastasis in models; (E, F) The number of metastatic nodules in the lung. Abbreviation: CD, chow diet; HFD, high-fat diet; TCA, taurocholic acid; *i.p.*, intraperitoneal injection.

Figure S2

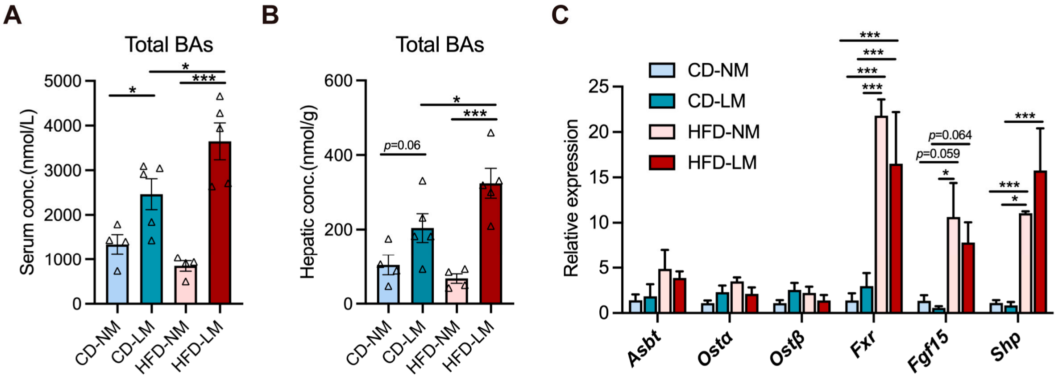

Figure S2. Serum and liver total BA levels and ileal metabolic gene expressions in mice fed CD or HFD. (A, B) The total BA levels in serum and liver; **(C)** The relative mRNA expressions of regulators to ileal BAs metabolism. The comparisons of total

BAs were determined with the one-way ANOVA method and BA-related genes among groups were analyzed by the two-way ANOVA test. Significance was shown as *, $p<0.05$; **, $p<0.01$; ***, $p<0.005$. Abbreviation: BA, bile acids; CD/HFD-NM, CD or HFD-fed mice without metastasis; CD/HFD-LM, CD or HFD-fed mice with liver metastasis; Asbt, apical sodium-bile acid transporter; Ostα, organic solute transporter α; Ostβ, organic solute transporter β; Fxr, farnesoid X receptor; Fgf15, fibroblast growth factor 15; Shp, small heterodimer partner.

Figure S3.

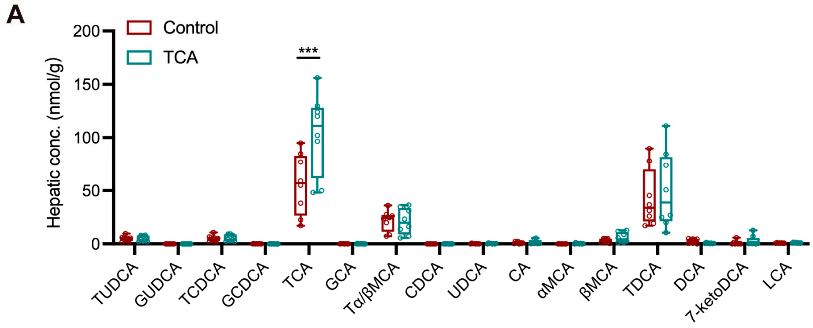

Figure S3. Hepatic BAs profiles in the TCA-treated mouse. (A) Concentrations of conjugated and free BA species in mouse liver. Comparisons of BA metabolites were determined with a one-way ANOVA test, significance was shown as ***, $p<0.005$.

Figure S4.

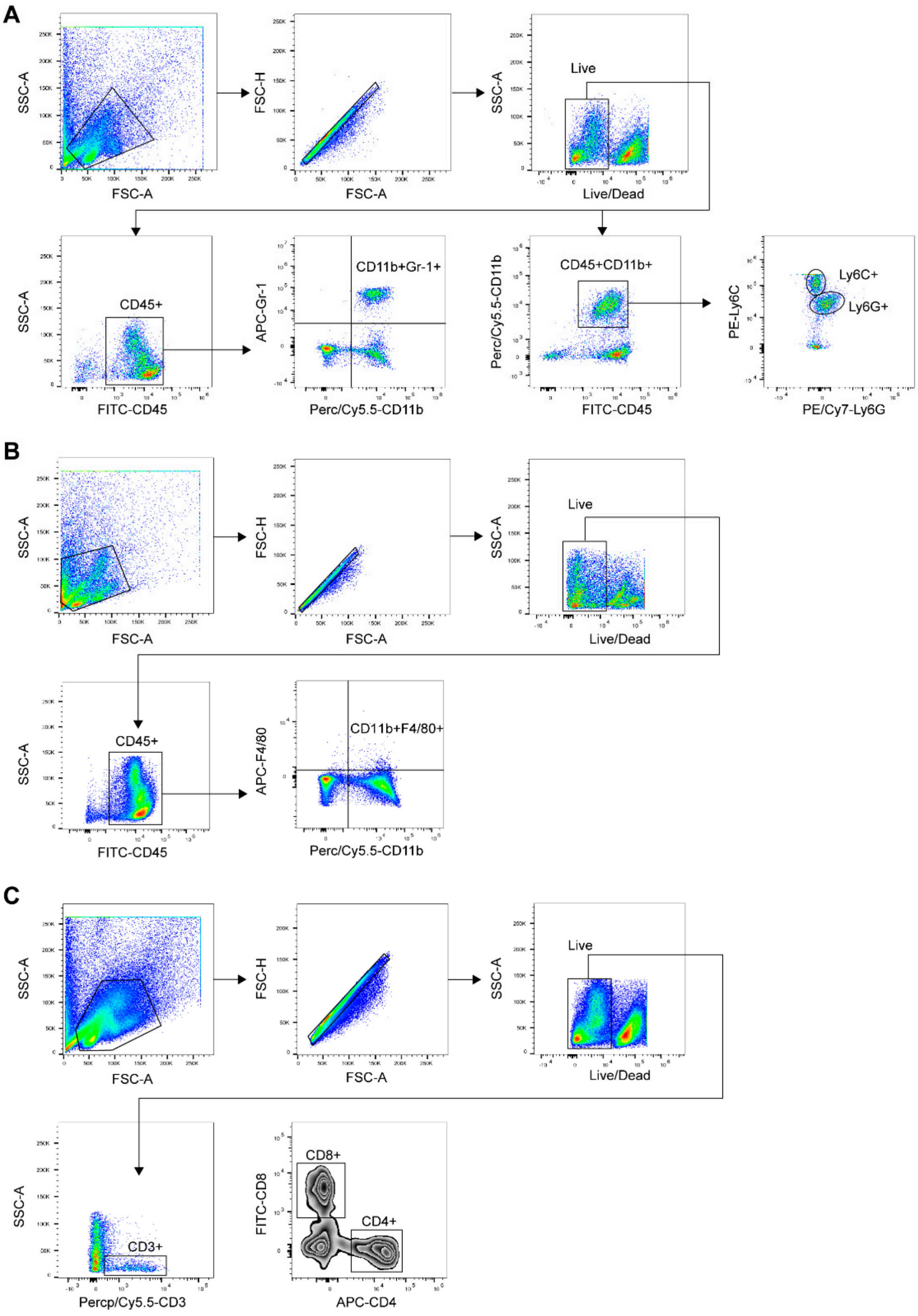

Figure S4. Gating strategy for immune cell subsets. (A) Gating strategy for identification of Gr-1^{+}, Ly6G^{+}, Ly6C^{+} cells; **(B)** Gating strategy for identification of F4/80^{+} cells; **(C)** Gating strategy for identification of CD3^{+}, CD4^{+}, CD8^{+} cells.

Figure S5

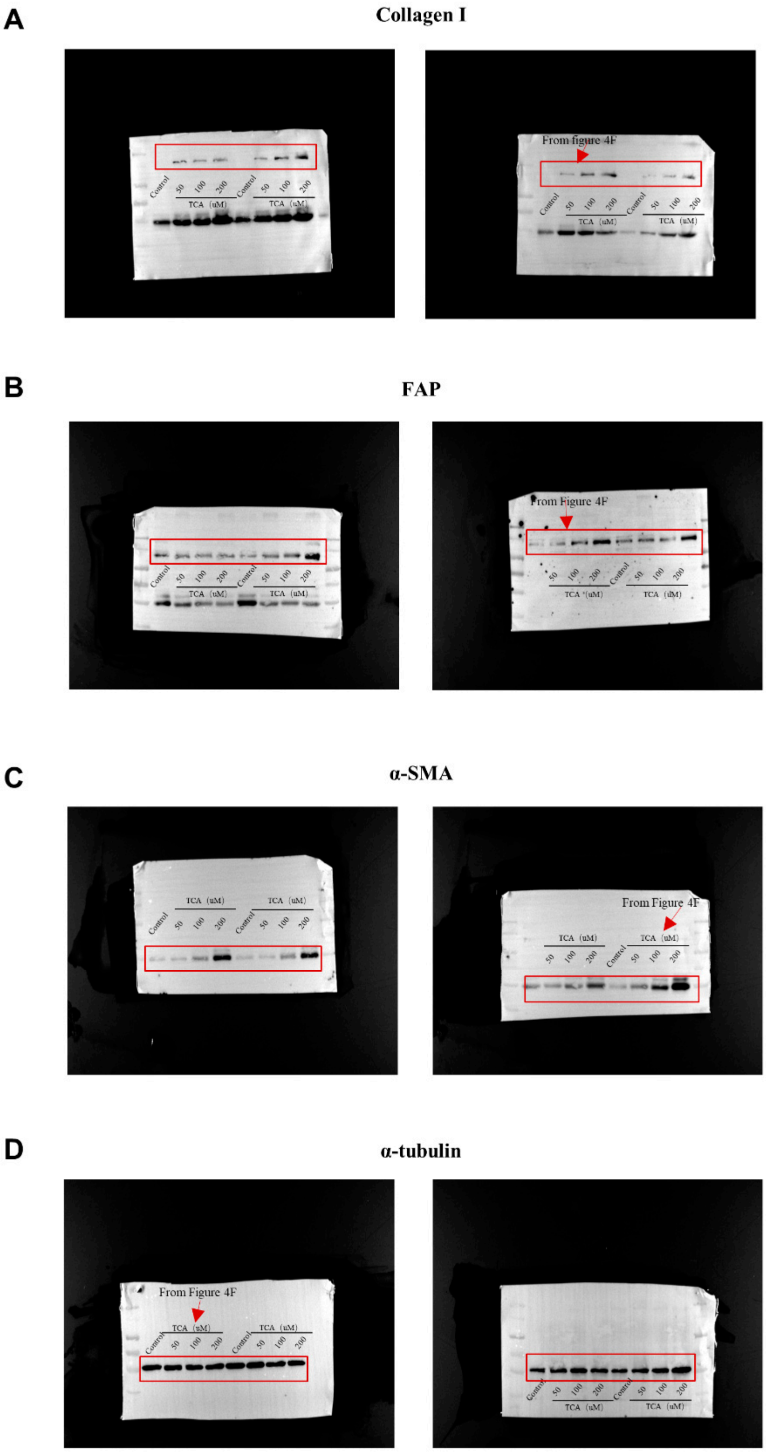

Figure S5. Uncropped western blots from main figures. Shown are uncropped blots from Figure 4F. The cropped region is pointed with the red arrow. **(A)** Uncropped blots

of Col I; **(B)** Uncropped blots of FAP; **(C)** Uncropped blots of α-SMA; **(D)** Uncropped blots of α-tubulin. Abbreviation: Col I, Collagen I; FAP, fibroblast activation protein; αSMA, alpha-smooth muscle actin.
